# Comprehensive Prognostic Analysis of Immune Implication Value and Oxidative Stress Significance of NECAP2 in Low-Grade Glioma

**DOI:** 10.1155/2022/1494520

**Published:** 2022-12-07

**Authors:** Zhichao Lu, Yixun Chen, Siqi Chen, Xingjia Zhu, Chenxing Wang, Ziheng Wang, Qi Yao

**Affiliations:** ^1^Department of Neurosurgery, Affiliated Hospital of Nantong University, Medical School of Nantong University, Nantong 226001, China; ^2^Research Center of Clinical Medicine, Affiliated Hospital of Nantong University, Medical School of Nantong University, Nantong 226001, China; ^3^Department of Clinical Biobank & Institute of Oncology, Affiliated Hospital of Nantong University, Medical School of Nantong University, Nantong 226001, China; ^4^Centre for Precision Medicine Research and Training, Faculty of Health Sciences, University of Macau, Macau SAR, China

## Abstract

Adaptin ear-binding coat-associated protein 2 (NECAP2) belongs to the family of proteins encoding adaptin-ear-binding coat-associated proteins. However, its immune effect on tumors and its microenvironment are still unclear. Here, we systematically evaluated the differences (variations) in NECAP2 expression for low-grade glioma (LGG) and pan-cancer in the LGG dataset of The Cancer Genome Atlas (TCGA) utilizing bioinformatics methods. We found for the first time that NECAP2 level was elevated in gliomas and that this upregulation increased as the tumor grade increased. In addition, Pearson correlations of NECAP2 with five immune pathways and significant gene mutations associated with NECAP2 were also analyzed. Univariate survival and multivariate Cox analyses were used to compare the clinical characteristics and survival of the patients. Glioma patients with NECAP2 overexpression have a remarkably higher risk of developing malignant behavior and a worse prognosis. The correlation between the expression levels of NECAP2 and the prognosis of glioma patients was identified. Kaplan-Meier curves showed that patients with upregulated NECAP2 expression exhibited an unfavorable prognosis. Western blotting showed that NECAP2 was overexpressed in glioma patients. IHC staining results illustrated an elevation in the NECAP2 protein expression level with the development of tumor malignancy. Additionally, qRT-PCR verified that oxidative stress in glioma tissues reduced the expression of stress-related genes and oxidative stress capacity compared to normal tissues, which may be associated with tumor evasion of immune surveillance and tumor progression. *In vitro* wound-healing and Transwell assay confirmed that NECAP2 promotes glioma cell migration and invasion. Our study also thoroughly examined the immune significance of NECAP2 in the TCGA-LGG samples, using CIBERSORT and ESTIMATE to explore the correlation between NECAP2 and cancer immune infiltration. The NECAP2 expression levels were correlated with the infiltration degree of immune cells such as neutrophils, CD4^+^ T cells, macrophages, CD8^+^ T cells, and B cells. Therefore, our results indicate that NECAP2 strongly correlates with the overall immune infiltration level of glioma and could independently serve as a prognostic biological marker for glioma patients.

## 1. Introduction

Gliomas are one of the most prevailing brain malignancies in adults, with their incidence rising worldwide [1]. Low-grade glioma (LGG) is one of the most pathogenic gliomas, with high invasiveness, diffuse infiltration, aggressive diffusion, and blurred boundaries [2, 3]. In the revised version of the World Health Organization's (WHO) classification of malignancies affecting the central nervous system (CNS) [4, 5], gliomas are classified based on combined molecular and histological findings [6], which include isocitrate dehydrogenase (IDH) mutation, 1p19q codeletion, H3 Lys27Met, and RELA fusion. Despite the development of molecular biology techniques, modern methods for pathogenesis, and diverse treatments of glioma [7], it is still a fatal disease with a very poor prognosis [8] and a mortality rate close to 80% in the first year of diagnosis. Through an excessive generation of reactive oxygen species (ROS), oxidative stress (OS) is a significant contributor to the onset and progression of cancers. Oxidative stress has been demonstrated to be implicated in a wide range of diseases including atherosclerosis, chronic obstructive pulmonary disease (COPD), Alzheimer's disease, and cancer, revealing multiple mechanisms by which oxidants contribute to cellular injury. Related basic studies have shown that an imbalance of intracellular oxidative stress is present in nearly all tumor cells. A prolonged high level of imbalance in the redox system directly leads to tissue damage and, on the other hand, leads to DNA mutations and protein spatial structure changes, which contribute to cancer development [9–11]. Recent research has provided strong evidence that OS is intimately linked to the advancement of LGG [12, 13]. However, the role of these OS-related genes in the prognosis of LGG and their underlying mechanism is still unclear. As a result, it is essential to make further progress in developing efficient treatment strategies to enhance the responsiveness of glioma patients against the detremental effects of oxidative stress.

PD-1 and PD-L1 inhibitors have promising therapeutic effects in Hodgkin lymphoma (HL) and malignant melanoma, bringing hope to better the treatment model of these diseases. However, the clinical effectiveness of existing immunotherapies for LGG has been subpar. The absence of indicators to guide personalized immunological targets is perhaps one of the factors contributing to therapy's ineffectiveness. For immunotherapy to be effective, tumor cells need to interface with their surrounding microenvironment. In particular, tumor-infiltrating neutrophils (TINs), and tumor-associated macrophages (TAMs) [14], impact the prognosis and effectiveness of chemotherapeutics and immunotherapeutic treatments [15, 16]. Immune infiltration into the tumor microenvironment (TME) correlates favorably with overall survival in LGG patients [17]. Therefore, it is imperative to determine novel immune-associated biological markers and therapeutic targets for glioma and to shed light on the immunophenotype of tumor-immune interactions.

In humans, adaptin ear-binding coat-associated protein 2 (NECAP2) is involved in endocytosis [18] and encode a member of the adaptin ear-binding coat-associated protein family [19]. NECAP2 is a pathway-specific modulator of clathrin coat formation on early endosomes, necessary for rapid endocytic cycling, maintenance of receptor levels on the cell surface, resensitization of cells to extracellular ligands, and sustained nutrition uptake [20]. NECAP2 regulates the endocytic cycle of EGFR and transferrin receptors [20, 21]. However, it has not been established whether NECAP2 has a role in the onset and advancement of malignancies, particularly in immune infiltration [22]. Therefore, we sought an in-depth comprehension of NECAP2 level in gliomas and its association with malignant characteristics and glioma patients' prognoses. Furthermore, to examine the predictive effectiveness of NECAP2 for high infiltration levels in gliomas, we examined the correlation between NECAP2 and infiltration degrees of various immune cells.

Consequently, we examined the link between NECAP2 expression in glioma, tumor-infiltrating immune cells (TIICs), and prognosis. We used Biodatabase to determine that glioma tissues express NECAP2 and screened the NECAP2 coexpressed genes related to oxidative stress. Kaplan-Meier analysis assessed the clinical significance of NECAP2 expression. Univariate and multivariate Cox regression analyses showed that NECAP2 independently served as a predictive biological marker for glioma. We then evaluated the link between NECAP2 expression levels and the level of TIICs in glioma tissues. Receiver operating characteristic (ROC) analysis was employed to verify the discriminative power of NECAP2 for LGG, its ability to predict poor prognosis in each clinicopathological subtype, and the predictive ability of other common immune-related markers. Importantly, NECAP2 overexpression is linked to the immune infiltration level of T cells, mast cells, neutrophils, NK cells, DCs, cytotoxic cells, DC, eosinophils, macrophages, Treg, and other immune cells and is intimately linked to the overall immune infiltration level of glioma. Based on these findings, NECAP2 seems to be a promising candidate biomarker for assessing the prognosis of glioma and immune infiltration.

## 2. Materials and Methods

### 2.1. Data Acquisition and Processing

The Cancer Genome Atlas (TCGA) was used to obtain the public data for this investigation. The RNA-seq profiles of TCGA-LGG samples and the relevant clinical data were acquired from the UCSC online platform (https://xenabrowser.net). For the subsequent analyses, patients with complete transcriptomic data and information on overall survival were selected.

### 2.2. Expression Levels and Correlation Analysis of Pan-Cancer

After obtaining the unified and standardized pan-cancer dataset from the UCSC database (https://xenabrowser.net/), we extracted the NECAP2 gene expression data of each sample in that dataset [23]. We simultaneously obtained DNAss tumor stemness score and microsatellite instability (MSI) [24] score by calculating the methylation characteristics of each tumor and then integrated the stemness index of the samples, MSI and gene expression data. The single-nucleotide variation dataset at level 4 of all TCGA samples was downloaded and processed with MuTect2 software from GDC (https://portal.gdc.cancer.gov/) for gene mutation analysis data and protein domain information.

### 2.3. Protein-Protein Interaction (PPI) and Enrichment Analysis

Based on previous studies, the STRING database (https://string-db.org/), an online resource, examined the interactions among proteins [25]. The STRING 11.0 version evaluated the PPI network between NECAP2 and the corresponding immunoregulators in the TCGA-LGG cohort [26]. The value of “0.4” was chosen as the confidence level. Gene Ontology (GO) [27] and Kyoto Encyclopedia of Genes and Genomes (KEGG) [28] enrichment analyses were performed to examine the biological roles and pathways related to these immunoregulators. A false discovery rate (FDR) <0.05 was considered statistically significant.

### 2.4. Evaluation of Patient Characteristics

We detected the diagnostic value for LGG from the TCGA database based on the NECAP2 expression. ROC curves were generated to estimate the biomarkers for predicting patient survival [29]. “rms” and “foreign” accessed the nomogram survival probability plot based on multivariate logistic regression. In addition, the index of concordance (c-index) was examined, and the nomogram estimates of survival probability were compared with the estimates from the Kaplan-Meier analysis.

### 2.5. NECAP2 Gene Set Enrichment Analysis (GSEA)

The GSEA computational method [30] was applied to determine the statistical significance of preferentially enriched gene sets and ascertain whether there were consistent variations across the two biological states. A thousand different permutations were performed on each gene combination that was investigated. The NECAP2 expression level was used as the phenotypic marker. A gene set with the identifier “c2.cp.v7.2.symbols.gmt” was obtained from a database of molecular markers, and the potential enrichment pathways were analyzed with GSEA (v 4.0.3). In addition, normalized *p* values, normalized enrichment scores (NESs), and FDR-adjusted *p* values (*q*-values) of GSEA were derived by segregating the enrichment pathways into 2 phenotypes. Gene sets with a q − value < 0.25 and a normalized *p* − value < 0.05 were considered significantly enriched.

### 2.6. Analysis of the Relative Abundance of Tumor-Infiltrating Immune Cells

A deconvolution technique, CIBERSORT (http://cibersort.stanford.edu/) [31], was utilized on gene expression to ascertain the relative correlation between NECAP2 expression and TIICs, and to characterize the cellular composition of complex tissues. The immune responses of 22 TIICs were assessed using CIBERSORT to determine the link between the NECAP2 low- and high-expression groups. The *p* value for each sample was then calculated according to the deconvolution algorithm.

### 2.7. Immunohistochemistry (IHC) Staining

After deparaffinization and dehydrating the tissue sections, they were subjected to epitope retrieval, treated with H_2_O_2_, and blocked against nonspecific bindings. The tissues were then incubated overnight with polyclonal rabbit anti-human NECAP2 antibodies (1 : 200, Proteintech, 15899-1-AP) at 4°C. Subsequently, the tissue sections were incubated with secondary antibodies (1 : 1000, Proteintech, SA00001-2) for two hours at ambient temperature. The signal was detected with an enhanced DAB staining kit (Proteintech, China).

### 2.8. Western Blotting

Tumor and normal tissues were lysed in RIPA buffer (Solarbio, Beijing, China), after which phosphatase and protease inhibitors were added and denatured for 15 minutes at 100°C. The protein samples were extracted with SDS-PAGE at a concentration of 10%, and the separated proteins were transferred into polyvinylidene fluoride (PVDF) membranes. The membranes were then blocked with a solution of skim milk powder at a concentration of 5% for 1 hour and incubated with primary antibodies, including anti-NECAP2 antibody (1 : 200, Proteintech, 15899-1-AP), anti-GAPDH antibody (1 : 5000, Abcam, ab9485) overnight. After thorough washing, the membranes were incubated with secondary antibodies (1 : 2000, Proteintech, SA00001-2) for 2 hours at ambient temperature and observed with the ECL kit chemiluminescence reagent (Billerica Millipore, MA, USA). The Chemidoc detection system (Bio-Rad, Hercules, California, USA) was employed to identify protein band signals. The ImageJ program (National Institutes of Health, USA) was used to quantify these signals.

### 2.9. Quantitative Reverse Transcription-Polymerase Chain Reaction (qRT-PCR)

Total RNA was extracted from normal tissue and tumor tissue from glioma patients using TRIzol reagent (Sigma-Aldrich, St. Louis, MO, USA). Quantitative reverse transcription-polymerase chain reaction (qRT-PCR) was conducted on the obtained RNA from each sample (2 *μ*g) with FastStart Universal SYBR ®Green Master (Roche, USA) on a LightCycler 480 PCR System (Roche, USA). The cDNA was utilized as a template with a reaction volume of 20 *μ*l (2 *μ*l of cDNA template, 10 *μ*l of PCR mixture, 0.5 *μ*l of forward and reverse primers, and an appropriate water volume). The following procedures were utilized for the PCR reactions: cycling conditions started with an initial DNA denaturation phase at 95°C for 30 seconds, followed by 45 cycles at 94°C for 15 seconds, 56°C for 30 seconds, and 72°C for 20 seconds. Three separate analyses were performed on each sample. Based on the 2^-*ΔΔ*CT^ method, data from the threshold cycle (CT) were obtained and standardized to the levels of glyceraldehyde 3-phosphate dehydrogenase (GAPDH) in each sample. The expression levels of mRNA were compared to controls obtained from normal tissues. The following is a list of the sequences of primer pairs for the genes that were being targeted Table 1:

### 2.10. Cell Culture and Transient Transfection

ATCC (Beijing Beina Chuanglian Biotechnology Institute) provided U-87 and U-251 human glioma cell lines, which were then incubated in F12 and DMEM supplemented with 10% fetal bovine serum (FBS, Gibco, Carlsbad, CA, USA), respectively. Both cell lines were kept in a humidified incubator and maintained at 37°C with 5% carbon dioxide. The negative control (NC) and NECAP2 siRNA (Sigma-Aldrich, St. Louis, MO, USA) were transfected into the glioma cells utilizing Lipofectamine 2000 (Invitrogen, Carlsbad, CA, USA) according to the manufacturer's guidelines. The target sequences for NECAP2 siRNAs were CAACATCGCAAACATGAAGAA (NECAP2 si 1) and GATGCCTTTGACTTCAATGTT (NECAP2 si 2). At the same time, cell culture dishes/plates, and centrifuge tubes were obtained from NEST Biotechnology Co. Ltd.(Wuxi, China).

### 2.11. Enzyme-Linked Immunosorbent Assay (Elisa)

The TGF-*β*1 protein levels (Sabbiotech, College Park, Maryland, USA) in the tissues or cultured cells were quantified by specific ELISA kits according to the manufacturer's guidelines.

### 2.12. Wound Healing and Transwell Assay

Monolayer glioma cells were inoculated in six-well plates and then scraped using sterile 200 *μ*l pipette tips before incubation in a serum-free medium (SFM). The width of the wound was recorded before and after 24 hours. Transwell assays for glioma cell (U-87, U-251) migration and invasion were performed. Briefly, cells (5 × 10^4^) were inoculated into chambers coated (for invasion) or uncoated with Matrigel (for migration; BD Biosciences, San Jose, CA). The top layer was added with SFM, whereas the bottom layer was added with a medium entirely composed of DMEM. Following an incubation period of 24 hours, migrating or invading cells were dyed with 0.1 percent crystalline violet and subsequently fixed with 4% paraformaldehyde. The counting of the cells was done under a light microscope.

### 2.13. Statistical Analysis

R software (v.3.6.1) was utilized to execute all statistical data analyses. The median value of NECAP2 expression was used as the cutoff value. The chi-PCR method, chi-square, and Fisher exact tests were employed to examine the clinicopathological parameters of the NECAP2 low- and high-expression groups. In addition, the Wilcoxon signed-rank test examined the degree of correlation between NECAP2 levels and clinicopathological parameters. Kruskal-Wallis test was used to compare multiple groups, including pathological grade, clinical stage, and tumor stage. We employed the Cox regression and Kaplan-Meier technique to measure Overall Survival in TCGA patients to determine the correlation of NECAP2 levels with patients' chances of survival and the clinicopathological variables. A *P* − value < 0.05 was considered statistically significant.

## 3. Results

### 3.1. Pan-Cancer Expression Level of NECAP2

**Figure 1:**
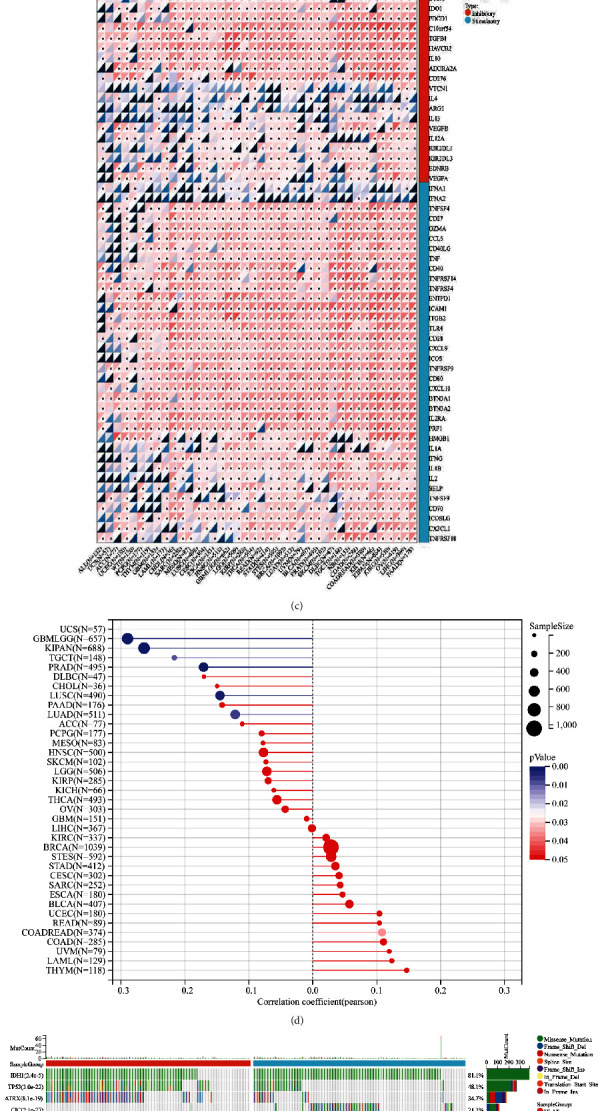
Pan-cancer expression levels of NECAP2. (a) Box plots showing the differential expression of NECAP2 between LGG and normal tissues. (b) Dot-line plots illustrating pan-cancer differential expression of NECAP2 in paired normal samples of LGG tissue and its corresponding patients. (c) Heatmap of Pearson correlations between NECAP2 and markers of the immune response. (d) Lollipop graph showing significant pan-cancer differences in NECAP2 expression. (e) Top15 related to NECAP2 in the TCGA-LGG dataset with significant gene mutation waterfall plot of mutation frequency differences.

We evaluated NECAP2 mRNA levels in pan-cancer tissues using the clinical data of 528 LGG patients acquired from the TCGA-LGG database. NECAP2 was found to be overexpressed in LGG tissues (Figure 1(a)). Pan-cancer differential expression analysis indicated that compared with paired normal tissue samples, NECAP2 mRNA levels in tumor tissue samples were significantly different in BLCA, CHOL, THCA, PRAD, LUSC, LUAD, LIHC, KIRP, STAD, KIRC, KICH, and HNSC (Figure 1(b)). The significant differences in NECAP2 in pan-cancer are shown in a lollipop plot (Figure 1(d)). Combined with marker genes in The Immune Landscape of Cancer, we analyzed the Pearson correlations between NECAP2 and five immune pathways, and the correlation heat map suggested significant correlations (Figure 1(c)).

Gene mutation is often a key link in tumor progression, and we showed the Top 15 genes associated with NECAP2 expression in the TCGA-LGG dataset with significant differences in mutation frequency (Figure 1(e)), including *IDH1* (*p* = 2.4e − 5), *TP53* (*p* = 3.0e − 22), *ATRX* (*p* = 8.1e − 19), *CIC* (*p* = 2.1e − 27), *TTN* (*p* = 0.10), *FUBP1* (*p* = 1.1e − 11), *NOTCH1* (*p* = 4.1e − 5), *PIK3CA* (*p* = 0.17), *EGFR* (*p* = 3.0e − 3), *PTEN* (*p* = 6.5e − 3), *IDH2* (*p* = 3.6e − 4), *ZBTB20* (*p* = 3.0e − 3), *NIPBL* (*p* = 5.0e − 3), *KAT6B* (*p* = 0.04), and *USP11* (*p* = 0.04).

### 3.2. Multidimensional Correlation of NECAP2 in Pan-Cancer

**Figure 2:**
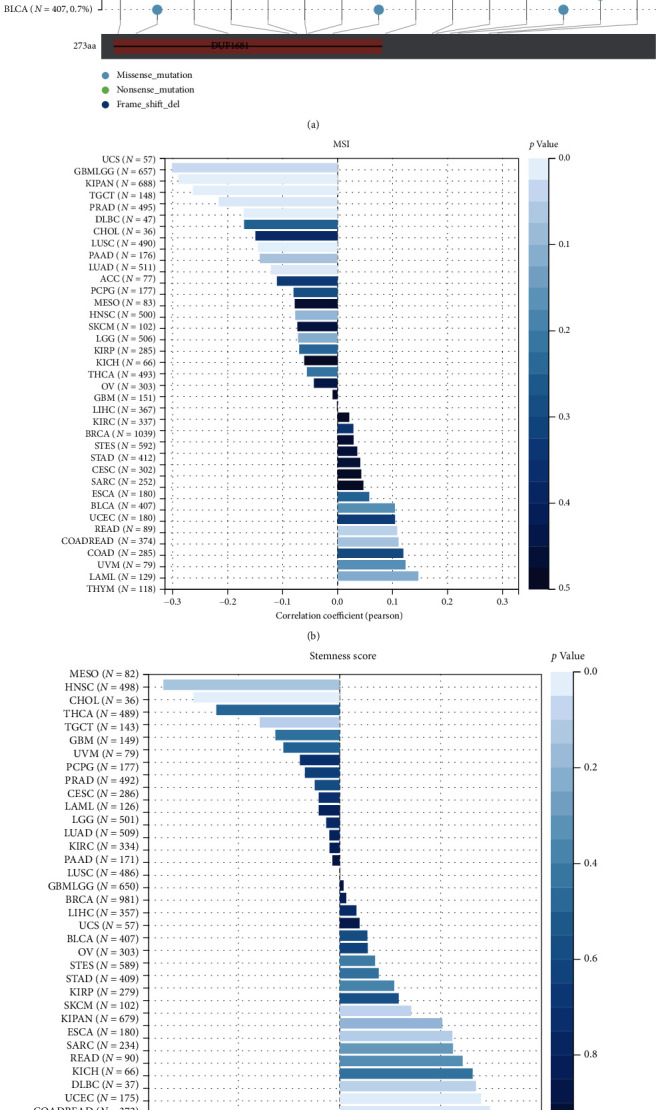
Multidimensional data analysis of NECAP2 in pan-cancer. (a) Domain information of mutant proteins of the samples. (b) Pan-cancer Pearson correlation of NECAP2 with MSI score showing a significant positive correlation in 1 tumor and negative correlation in 7 tumors. (c) The Pearson correlations of DNAs tumor stemness scores calculated based on NECAP2. Pan-cancer methylation signatures show significant positive correlations in 6 tumors and negative correlations in the rest of the tumors.

We integrated multidimensional data to analyze the pan-cancer function and significance of NECAP2. Firstly, the protein domain information was obtained from the mutation data of the samples, and by obtaining the DNAs tumor stemness score and Microsatellite instability (MSI) score calculated by each tumor methylation signature, the stemness index, MSI, and MSI scores of the samples were integrated. Pearson correlation of NECAP2 in pan-cancer was calculated from gene expression data. NECAP2 showed a significant correlation with MSI in 8 tumors, of which only 1 showed a positive correlation, COADREAD (*N* = 374, *r* = 0.108, Figure 2(a)). Rest 7 tumors showed a negative correlation, including GBMLGG (*N* = 657, *r* = −0.290), LUAD (*N* = 511, *r* = −0.121), KIPAN (*N* = 688, *r* = −0.264), PRAD (*N* = 495, *r* = −0.171), LUSC (*N* = 490, *r* = −0.145), TGCT (*N* = 148, *r* = −0.216), and UCS (*N* = 57, *r* = −0.301, Figure 2(b)). At the same time, tumor stem Sex index was significantly correlated in 15 tumors, of which 6 showed a positive correlation, such as: GBMLGG (*N* = 558, *r* = 0.367), LGG (*N* = 507, *r* = 0.312), CESC (*N* = 301, *r* = 0.119), LAML (*N* = 170, *r* = 0.193), SARC (*N* = 253, *r* = 0.131), and THCA (*N* = 499, *r* = 0.139). The remaining tumors showed a negative correlation (Figure 2(c)).

### 3.3. Construction of a PPI Network of Oxidative Stress-Related DEGs in LGG

**Figure 3:**
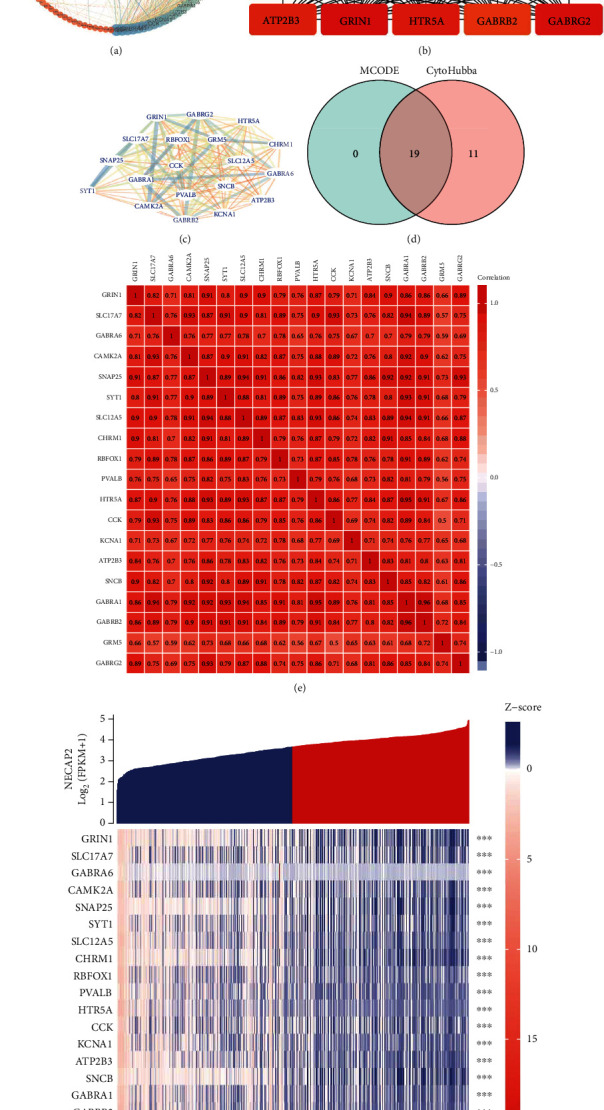
Development of a PPI network of DEGs related to oxidative stress. (a) A protein-protein interaction (PPI) network was created using the STRING database to represent the intermolecular interactions, and the maximum confidence interaction score was adjusted to 0.4, which was analyzed and visualized by Cytoscape's Network Analyzer tool. (b) Top 30 closely related genes were screened using the CytoHubba plugin. (c) The closely related genes of the PPI network modules were screened and visualized using the MCODE plugin. (d) A Venn diagram depicting the intersection of the two approaches shows 19 oxidative stress-related differentially expressed genes. (e) The correlation heat map between 19 closely related Hub genes. (f) The heat map of the differential expression of the Hub genes between low- and high-expression groups of NECAP2.

The STRING database was used to construct a PPI network to reflect intermolecular interactions, with a maximum confidence interaction score set at 0.4, which was analyzed and visualized by Cytoscape's Network Analyzer tool (Figure 3(a)). Top 30 closely related genes were screened using the CytoHubba plugin. At the same time, the closely related genes of the PPI network modules were screened and visualized using the MCODE plugin (Figures 3(b) and 3(c)). A Venn diagram depicting the intersection of the two approaches yielded 19 oxidative stress-related differentially expressed genes (Figure 3(d)). This included *GRIN1*, *SLC17A7*, *GABRA6*, *CAMK2A*, *SNAP25*, *SYT1*, *SLC12A5*, *CHRM1*, *RBFOX1*, *PVALB*, *HTR5A*, *CCK*, *KCNA1*, *ATP2B3*, *SNCB*, *GABRA1*, *GABRB2*, *GRM5*, and *GABRG2*. The heat map indicated a significant positive correlation between the 19 closely related Hub genes (Figure 3(e)). The heat map further demonstrated significant differential expression of the Hub genes between the low- and high-expression groups of NECAP2 (Figure 3(f)).

### 3.4. GO and KEGG Enrichment Analysis of Differentially Expressed Hub Genes of NECAP2

**Figure 4:**
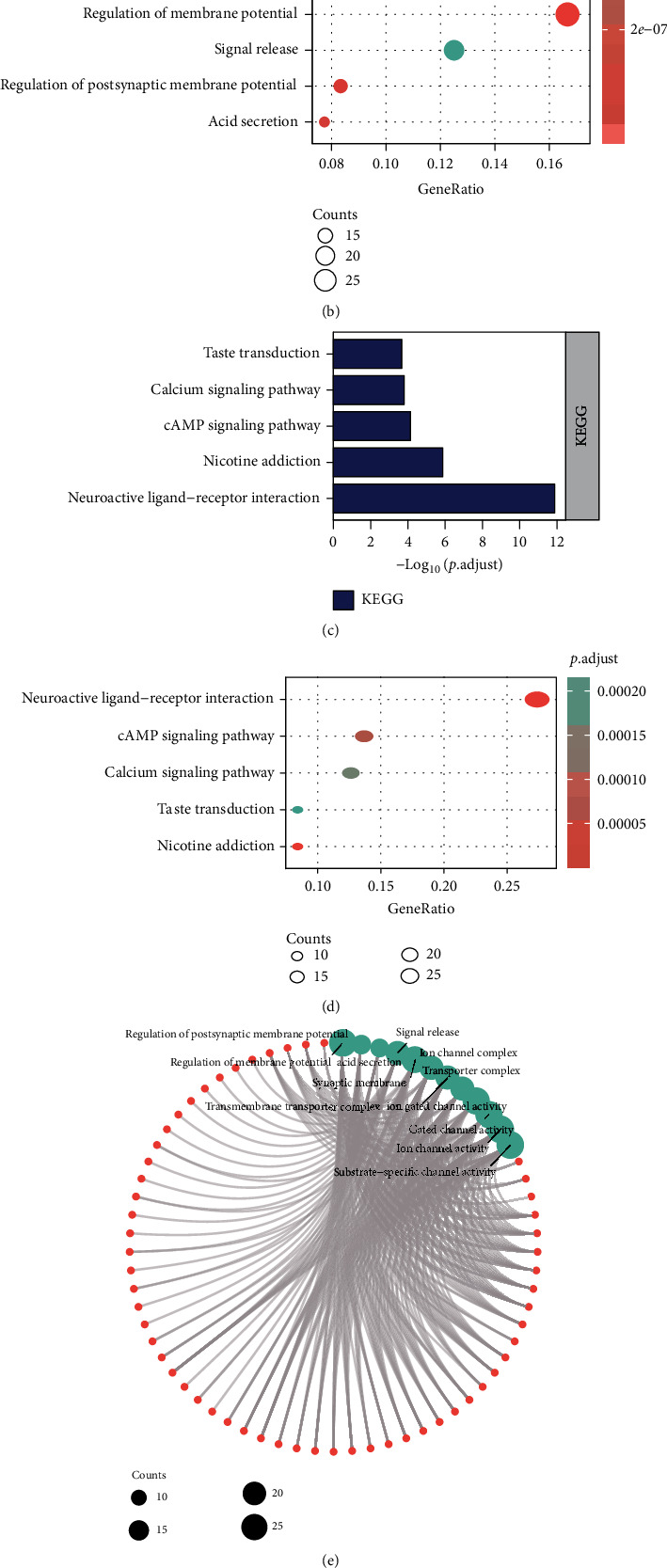
Enrichment analysis of hub genes. (a, b, and e) Histograms, bubble charts, and chord charts of GO enrichment analysis of differentially expressed genes in hubs of NECAP2. (c, d, and f) Histograms, bubble charts, and chord charts of KEGG enrichment analysis of differentially expressed genes in hubs of NECAP2.

We evaluated the individual contributions of hub coexpressed differentially genes of NECAP2 (Figures 4(a), 4(b), and 4(e)). The significant MF, CC, and BP GO terms comprised multiple metabolic and biosynthetic processes, signaling pathways, modulation of cell migration, modulation of postsynaptic membrane potential, transporter complex, acid secretion, transmembrane transporter complex, signal release, cognition, postsynaptic membrane, synaptic membrane, neurotransmitter receptor activity, ion channel complex, substrate-specific channel activity, ion channel activity, gated channel activity, and ion gated channel activity. The significant pathways comprised multiple metabolic processes and biosynthetic, signaling pathways, modulation of cell migration, calcium signaling pathway, cAMP signaling pathway, Nicotine addiction, neuroactive ligand-receptor interaction, and taste transduction (Figures 4(c), 4(d), and 4(f)). These enriched GO terms are represented as networks in Tables 2 and 3.

### 3.5. Identifaction of NECAP2-Related Signaling Pathways Using GSEA

**Figure 5:**
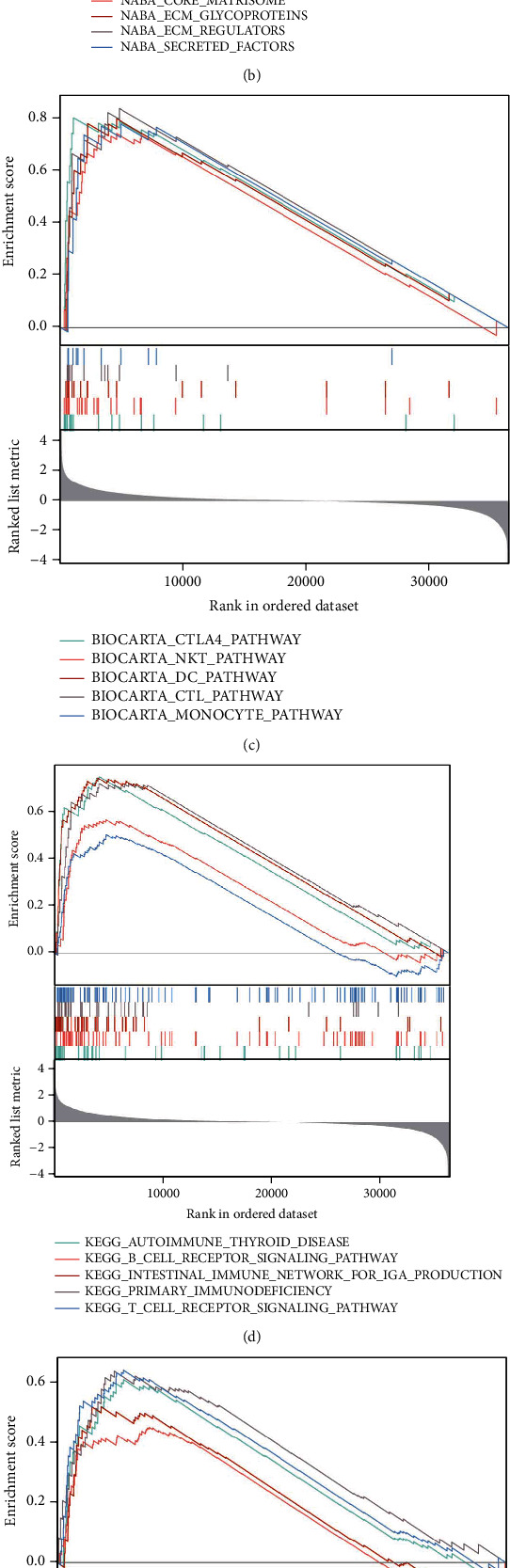
GSEA enrichment analysis of NECAP2. Enriched pathways in WP (a), NABA (b), BIOCARTA (c), KEGG (d), PID (e), and REACTOME (f) databases, respectively.

The WP database was enriched in cancer immunotherapy by pd-1 blockade, miRNAs involvement in the immune response in sepsis, human immune response to tuberculosis, retinoblastoma gene in cancer, senescence, and autophagy in cancer (Figure 5(a)). the NABA database was enriched in collagens, core matrisome, ecm glycoproteins and regulators, and secreted factors (Figure 5(b)). The biocarta database was enriched in ctla4, nkt, dc, ctl, and monocyte pathway (Figure 5(c)). The KEGG database was enriched in T cell receptor signaling pathway, primary immunodeficiency, intestinal immune network for IgA production, B cell receptor signaling pathway, and autoimmune thyroid disease (Figure 5(d)). the pid database was enriched in cd8 tcr, cxcr4, il2, il6, il7, and tcr pathway (Figure 5(e)). the reactome database was enriched in CD22 mediated BCR regulation, diseases of immune system, signaling by the B cell receptor BCR pathway, and immunoregulatory interactions between a lymphoid reactome pd-1 signaling (Figure 5(f) and Table 4).

### 3.6. Relationship between NECAP2 and Clinicopathological Features

The TCGA-LGG database was analyzed to obtain patients' clinical information.  Table 5 shows the relationship between NECAP2 expression and clinicopathological factors (TCGA-LGG), including WHO grade (G2 vs. G3, *p* = 0.017), IDH status (WT vs. Mut, *p* < 0.001 between high and low-NECAP2 expression groups), and 1p/19q codeletion (codel vs. noncodel, *p* < 0.001). Subsequently, primary therapy outcome (PR&CR vs. PD&SD), 1p/19q co-deletion (noncodel vs. codel), WHO grade (G3 vs. G2), histological type (Oligodendroglioma and Oligoastrocytoma versus Astrocytoma), laterality (Right vs. Left), and age (>40 vs. <=40), IDH status (Mut vs. WT), and gender (Female vs. Male) was included to the analysis. All the patients were classified into low and high-expression groups based on the median level of NECAP2 expression. The investigation revealed that the prognosis of LGG patients was highly influenced by factors such as the histological type, the 1p/19q co-deletion, WHO grade, and the status of IDH (Table 6).  Table 7 presents the findings of both univariate and multivariate Cox analyses. The association of NECAP2 expression with clinicopathological variables was summarized. Our results suggested that age, IDH status, primary therapy outcome, WHO grade, and NECAP2 expression might independently serve as prognostic indicators for LGG patients.

### 3.7. Differential Expression of NECAP2 in Pathological Subgroups in the TCGA-LGG Dataset

**Figure 6:**
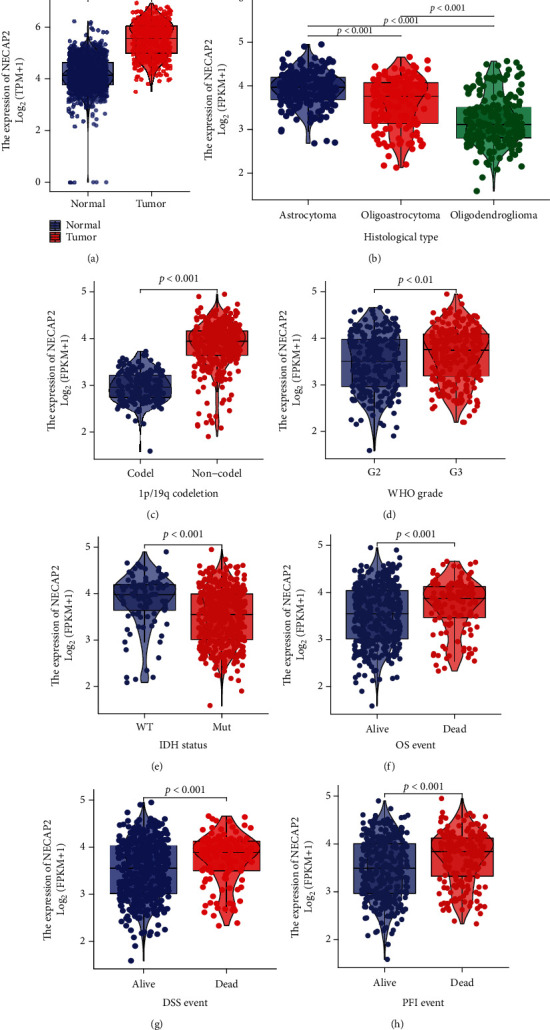
Association of NECAP2 expression with clinicopathological characteristics in the TCGA-LGG dataset. (a) Differences between LGG and normal samples. (b–h) Expression difference between subgroups of pathological parameters (*p* < 0.001).

**Figure 7:**
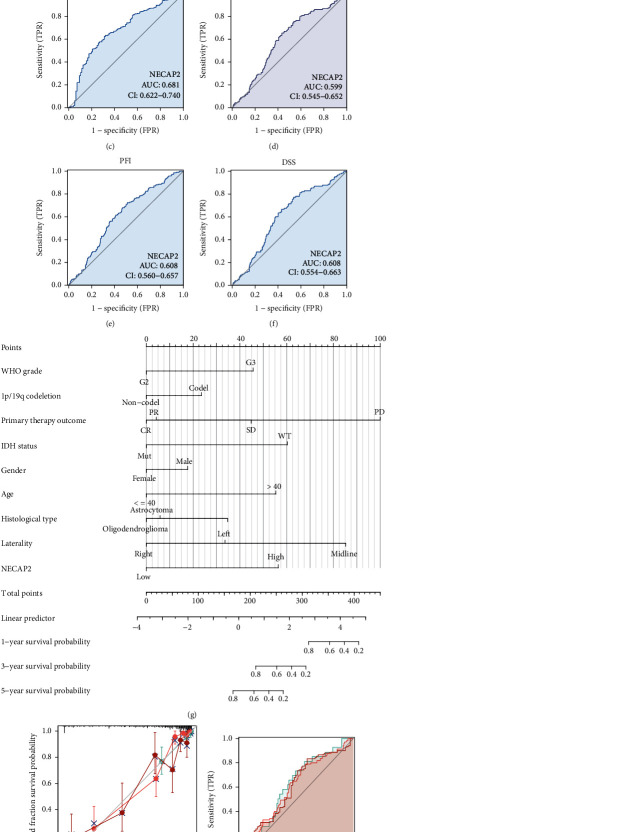
The predictive power of NECAP2 expression for prognosis of clinical case variables in LGG patients. (a) The AUC of NECAP2 in TCGA-LGG was 0.924. (b–f) NECAP2 shows good efficiency in distinguishing subgroups of clinicopathological variables, including 1p/19q codeletion, IDH status, OS, DSS, and PF prognosis. (g) Prediction of 1-, 3-, and 5-year survival rates in LGG patients using a nomogram. (h) Calibration curves for evaluating nomogram-predicted 1-, 3-, and 5-year survival to show the difference between nomogram prediction and actual survival. (i) The time-dependent ROC curve shows that NECAP2 has a good predictive ability for the survival prognosis of 1, 3, and 5 years LGG patients.

We analyzed the differences between LGG and normal samples and the expression differences between subgroups of pathological parameters (*p* < 0.001, Figure 6(a)), including IDH status (Mut vs. WT), histological type (Oligodendroglioma and Oligoastrocytoma versus Astrocytoma), 1p/19q codeletion (noncodel vs. codel), WHO grade (G3 vs. G2), OS (Alive vs. Dead), DSS (Alive vs. Dead), and PFI (Alive vs. Dead). The results showed a significantly higher expression in Astrocytoma, noncodel, G3, WT, and dead samples (Figures 6(b)–6(h)). Furthermore, the area under the ROC curve (AUC) of NECAP2 in TCGA was 0.924 (Figure 7(a)). AUC of the overall survival model illustrated that our model had outstanding effectiveness in predicting 1p/19q codeletion, IDH status, OS, DSS, and PF prognosis (Figures 7(b)–7(f)). A nomogram prediction model was generated by integrating the above clinicopathological parameters and CDKL2 expression levels (Figure 7(g)). To assess 1-, 3-, and 5-year survival rates by nomogram, we generated calibration curves (Figure 7(h)) to illustrate that the predictions made by the nomogram and actual survival have a considerable agreement. Furthermore, the time-dependent ROC curve showed that NECAP2 had a remarkable performance in predicting the survival prognosis of LGG in the 1^st^ year compared to 3^rd^ and 5^th^ years (Figure 7(i)).

### 3.8. Overall Survival Prognostic Analysis of NECAP2 in Pathological Subgroups

**Figure 8:**
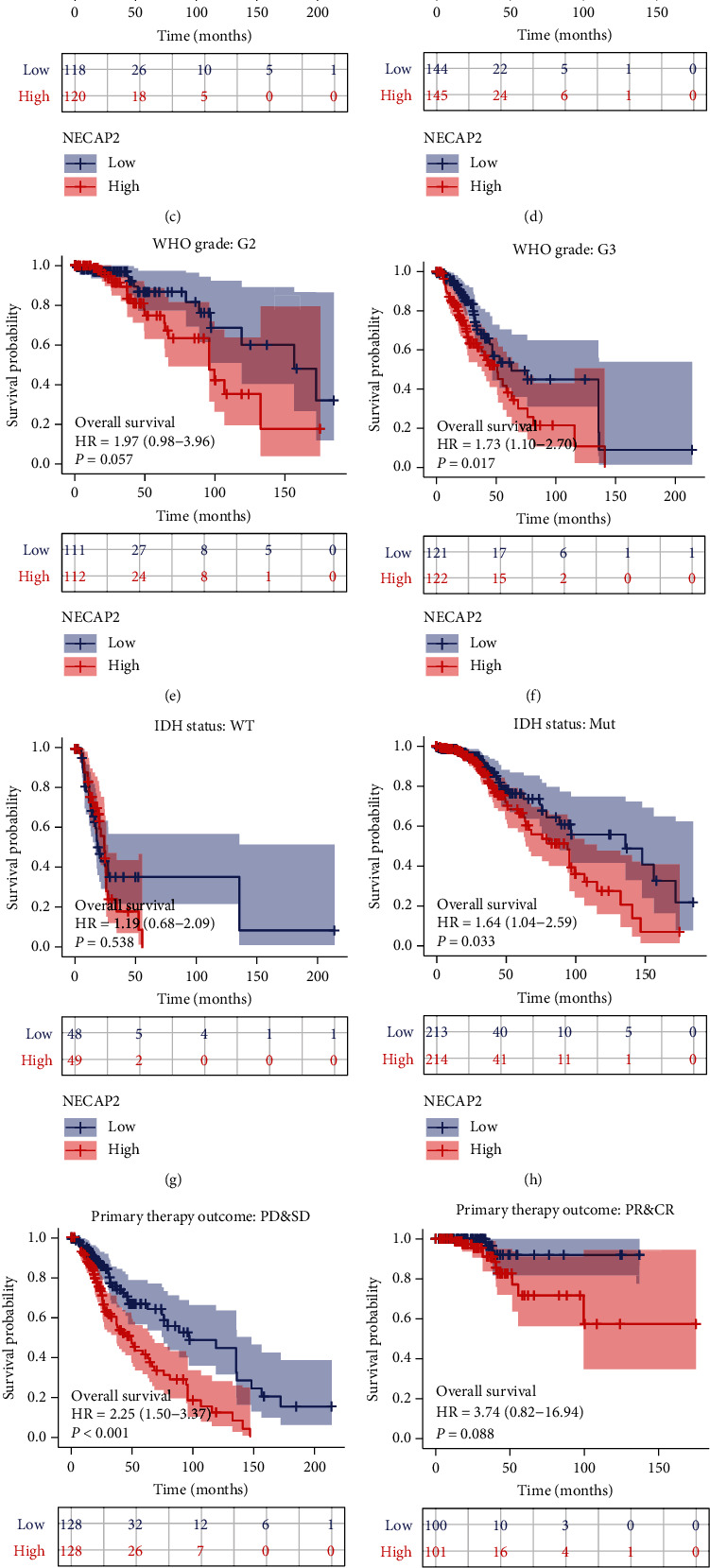
Kalpan-Meier curves of NECAP2 show the overall survival of LGG patients in each pathological subgroup in TCGA-LGG. (a, b) NECAP2 shows a significant overall survival prognosis in age> 40 and < =40 clinical subgroups. (c, d) NECAP2 shows a significant overall survival prognosis in both male and female clinical subgroups. (e, f) NECAP2 shows a significant overall survival prognosis in the G3 clinical subgroup but not in the G2 subgroup. (g, h) NECAP2 shows a significant overall survival prognosis in the Mut clinical subgroup but not in the PR and CR subgroup. (i, j) NECAP2 shows a significant overall survival prognosis in the PD and SD clinical subgroup but not in the WT subgroup. (k, l) NECAP2 shows a significant overall survival prognosis in the Oligoastrocytoma and Oligodendroglioma and clinical subgroups, but not in the Astrocytoma subgroup.

We subsequently carried out the overall survival prognostic analysis of LGG. Using the K-M curve, we demonstrated the overall survival prognosis difference across the low- and high-expression groups of NECAP2 in each pathological subgroup of TCGA-LGG samples. The OS time and prognosis for the patient were favorable, and statistical analysis results showed significant differences in most clinical subgroups. Our results suggested that the clinical subgroups of age (>40 and ≤40), gender (Male and Female), G3, Mut, PD&SD, Oligoastrocytoma and Oligodendroglioma have significant prognostic significance for OS (Figures 8(a)–8(d), 8(f), 8(h), 8(i), and 8(l)). However, there was no statistical significance in the G2, WT, PR&CR, and astrocytoma subgroups (Figures 8(e), 8(g), 8(j), and 8(k)).

### 3.9. Immune Cell Infiltration Analysis

**Figure 9:**
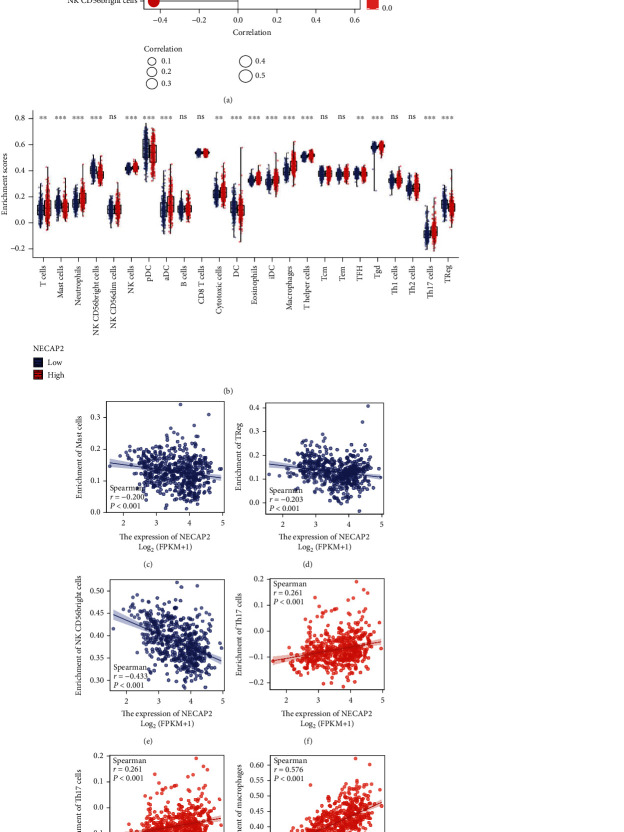
Analysis of immune cell infiltration. (a, b) Lollipop plot shows CIBERSORT analysis of NECAP2 and 22 distinct types of immune cell infiltration correlation and box plot shows the distribution difference. (c–h) The Scatter plot shows CIBERSORT analysis of NECAP2 and 22 different immune cell infiltration.

**Figure 10:**
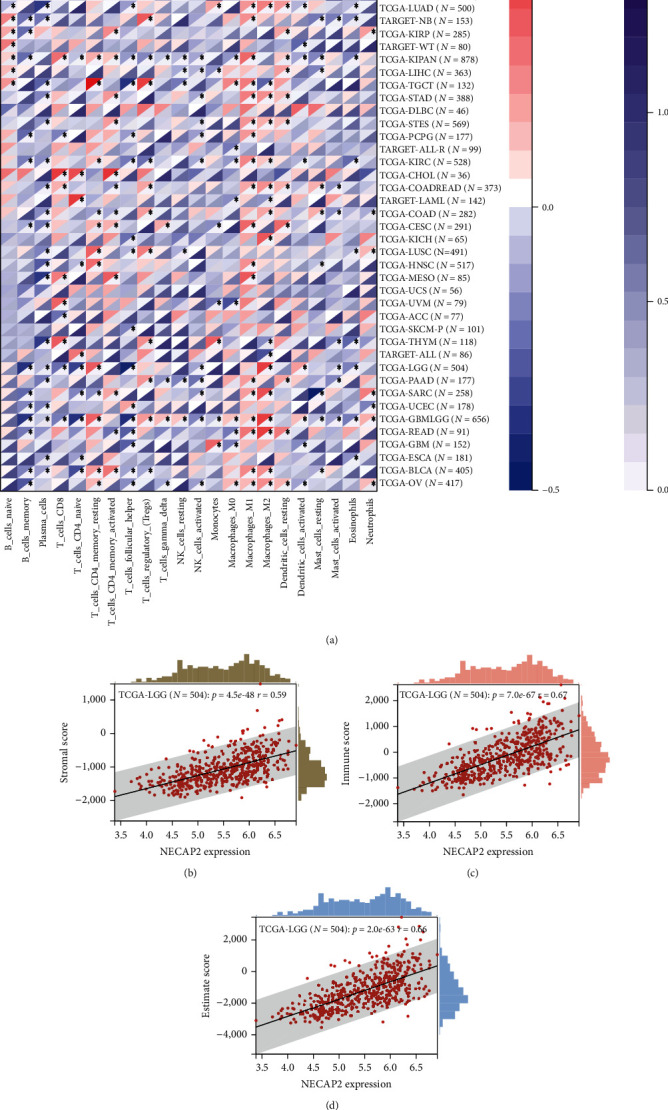
ESTIMATE correlation analysis of immune cell infiltration. (a) A heat map illustrating the distinct association of 22 different immune cell infiltration in pan-cancer. (b–d) ESTIMATE analysis of stromal cell score level and ESTIMATE score and stromal, respectively. Scatter plot of scores showing correlation with NECAP2.

CIBERSORT analysis of the link between NECAP2 and 22 different kinds of immune cell infiltration, lollipop plot and distribution difference box plot prompts, T cells, NK CD56bright cells, Neutrophils, NK cells, Mast cells, pDC, aDC, Cytotoxic cells, DC, and Eosinophils were evaluated. Significant differences were found in the infiltration level of iDC, macrophages, T helper cells, TFH, Tgd, Th17 cells, and Treg between the low- and high-expression groups of NECAP2 (Figures 9(a) and 9(b)). Scatter plots illustrated significant associations of NECAP2 with 22 types of immune cell infiltration in CIBERSORT analysis (Figures 9(c)–9(i)). A heat map of differential correlations in the infiltration of 22 distinct immune cells in pan-cancer was also analyzed (Figure 10(a)). Scatter plots suggested that ESTIMATE analysis of NECAP2 correlation scatter plots were positively correlated with tumor purity, immune score, estimate scores, and stroma scores (Figures 10(b)–10(d)).

### 3.10. Correlation of NECAP2 with Tumor Phenotypic Biomarkers

**Figure 11:**
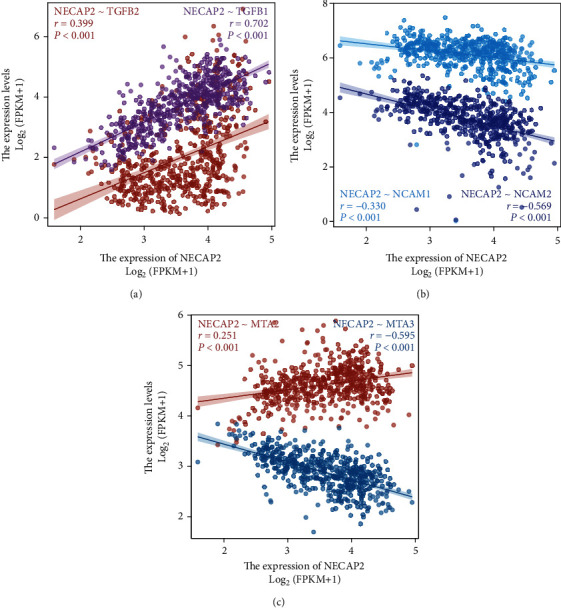
Correlation of NECAP2 with tumor phenotypic markers. (a) NECAP2 shows significant positive correlation with cell proliferation-related genes TGFB1 (r = 0.399) and TGFB2 (*r* = 0.702, *p* < 0.001). (b) NECAP2 negatively correlates to both the cell adhesion molecules NCAM1 (r = −0.330) and NCAM2 (*r* = −0.569, *p* < 0.001). (c) NECAP2 positively correlates to MTA2 (*r* = 0.251, *p* < 0.001), but correlates inversely to MTA3 (*r* = −0.595, *p* < 0.001).

NECAP2 showed significant positive correlation with both cell proliferation-related genes TGFB1 (*r* = 0.399) and TGFB2 (*r* = 0.702, *p* < 0.001, Figure 11(a)). NECAP2 showed significant negative correlation (*p* < 0.001) with both the cell adhesion molecules NCAM1 (*r* = −0.330) and NCAM2 (*r* = −0.569, Figure 11(b)). NECAP2 was also positively linked to MTA2 (*r* =0.251, *p* < 0.001), and negaively linked to MTA3 (*r* = −0.595, *p* < 0.001, Figure 11(c)).

### 3.11. A High Level of NECAP2 Is Associated with Glioma Progression and Reduced Oxidative Stress

**Figure 12:**
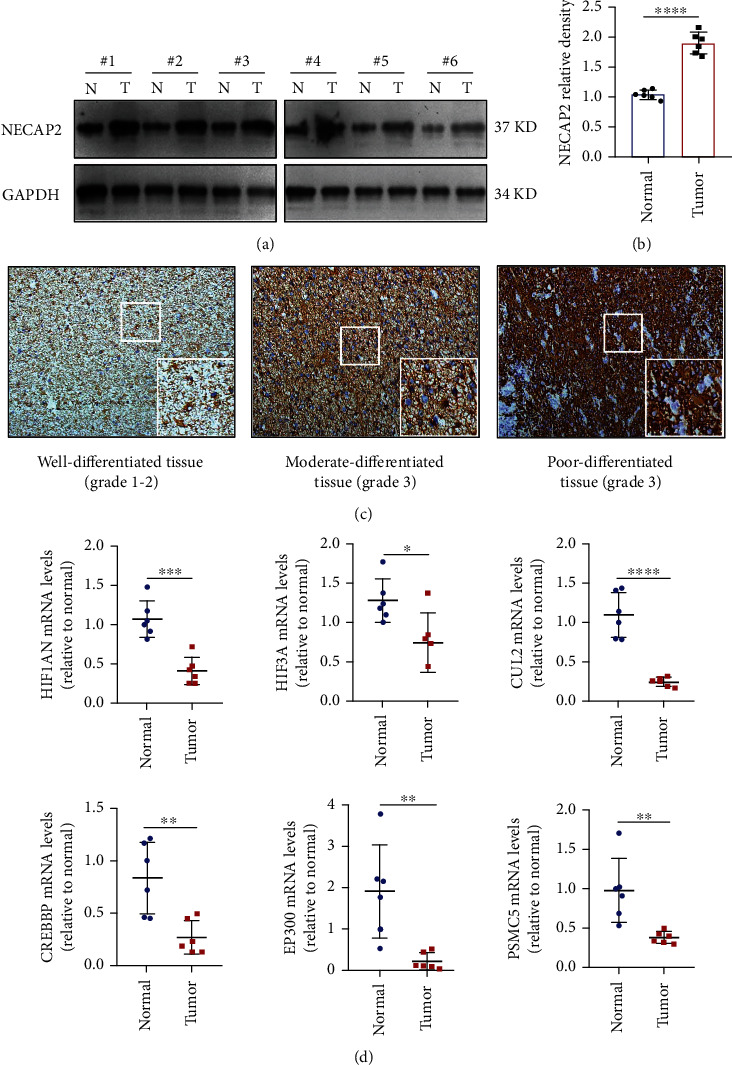
NECAP2 expression is upregulated in clinical glioma tissues with decreased expression of oxidative stress-related genes. (a, b) Protein levels of NECAP2 in six pairs of glioma tissues (T) and nearby nontumor tissues (N) as detected by western blotting. (c) Representative images of IHC staining of NECAP2 in glioma tissues of distinct histological grades (The overall magnification is 10X, and the local magnification is 40X). (d) Expression of oxidative stress-related genes in six pairs of glioma tissues (T) and nearby nontumor tissues (N). ^∗^*p* < 0.05, ^∗∗^*p* ≤ 0.01, ^∗∗∗^*p* ≤ 0.001, and ^∗∗∗∗^*p* ≤ 0.0001.

To assess the function of NECAP2 in glioma progression, we examined the NECAP2 protein expression utilizing western blotting and IHC staining (Figures 12(a)–12(c)). Western blotting showed that NECAP2 was overexpressed in glioma patients. IHC staining illustrated that the NECAP2 protein expression enhanced with the development of tumor malignancy (marked by changes in histological grade). To investigate whether the high expression of NECAP2 is associated with reduced oxidative stress capacity, we examined the mRNA expression of HIF1AN, HIF3A, CUL2, CREBBP, EP300, and PSMC5 by qRT-PCR (Figure 12(d)). Compared to normal tissues, the expression of oxidative stress-related genes was reduced in glioma tissues. The reduced oxidative stress capacity might be related to tumor evasion of immune surveillance and tumor progression.

### 3.12. NECAP2 Promotes Migration and Invasiveness of Glioma Cells

**Figure 13:**
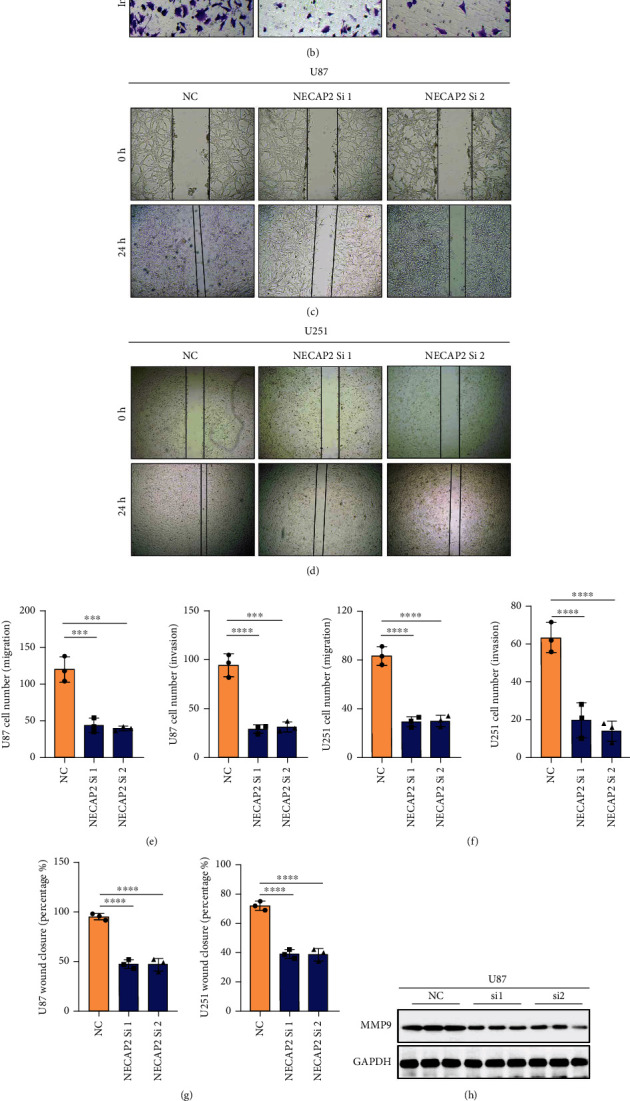
NECAP2 promotes glioma cell migratory and invasive capacity *in vitro*. (A-B) Images showing wound healing assay for negative control and NECAP2 knockout groups. (c, d) Transwell assay images of migration and invasion in the negative control and NECAP2 knockout groups. (e, f) Quantitative analysis of wound healing assays. (g) Quantitative analysis of migrating and invading glioma cells. (h, i) Expression of MMP9 in U87 and U251 cell lines after normal and interference. (j, k) TGF-*β*1 concentration in cell culture supernatants measured by ELISA in glioma cells transfected with siRNA. ^∗∗^*p* ≤ 0.01, ^∗∗∗^*p* ≤ 0.001, and ^∗∗∗^*p* ≤ 0.0001.

The transwell assay with NECAP2 knockdown cells revealed a reduced ability of cells to migrate and invade (Figures 13(a), 13(b), 13(e), and 13(f)). *In vitro* wound healing assays demonstrated that NECAP2 knockdown cause attenuation in the migratory capacity of the cells (Figures 13(c), 13(d), and 13(g)). The transwell assay with NECAP2 knockdown cells revealed a reduced ability of cells to migrate and invade (Figures 13(h) and 13(i)). TGF-*β*1 promotes the development of glioma through multiple pathways. So TGF-*β*1 is considered a key promoter of migratory and invasive properties of glioma cells. Interestingly, the secretion of TGF-*β*1 was reduced when NECAP2 was downregulated. These findings suggest that NECAP2 might be necessary for the migration and invasion of glioma cells *in vitro* (Figures 13(j) and 13(k)).

## 4. Discussion

LGG is a malignancy that poses a global risk to public health. Lack of early diagnosis and limitations of traditional treatment methods have led to unsatisfactory results and prognostic value [32]. Oxidative stress is an additional metabolic feature in the tumor microenvironment (TME) [33]. Cancer cells are well adapted to persistent oxidative stress through a series of mechanisms that are integral in both the activation of the ROS scavenging system and suppression of apoptosis [14]. Studies have shown that this adaptation has a role in the transition from benign to malignant during cancer metastases and the development of resistance to anticancer agents [34]. Therefore, understanding the mechanism behind ROS adaptation is of great significance for effectively destroying cancer cells and overcoming drug resistance [35]. In the present study, the expression level of NECAP2 was shown for the first time to be highly elevated in gliomas and increased with tumor grade [36]. From this, we analyzed the Pearson correlation of NECAP2 and five types of immune pathways. We integrated pan-cancer multidimensional data to analyze the correlation of NECAP2 with DNAs tumor stemness score and MSI score calculated from methylation signatures and significant gene mutations associated with NECAP2. The function and pathway enrichment of oxidative stress-related coexpressed genes of NECAP2 were also explored.

In the clinical prognosis analysis, the effect of NECAP2 on the OS of LGG patients was examined through the survival module, and the survival analysis of clinical subgroup variable samples was also performed. Both indicated that patients exhibiting elevated NECAP2 expression levels experienced a reduced OS time and poor prognosis. The correlation between clinical data and NECAP2 expression was evaluated using logistic regression. Univariate survival and multivariate Cox analyses were conducted to examine the differences between the clinical parameters and survival. NECAP2 overexpression was linked to malignant characteristics and unfavorable prognosis in glioma patients. We further investigated the link between NECAP2 expression and prognosis in glioma patients. The Kaplan-Meier survival curves demonstrated that patients with high NECAP2 levels had a worse prognosis. At the same time, in the LGG, IDH mutant, IDH wild-type, 1p/19q co-deletion, 1p/19q non-codeletion, young, and elderly subgroups, the prognosis of patients with NECAP2 overexpression was significantly worse in contrast with that of the low expression group. Western blotting showed that NECAP2 was overexpressed in glioma patients. The findings of the IHC staining confirmed that the expression of the NECAP2 protein was enhanced as the malignancy of the tumor increased. Additionally, qRT-PCR provided conclusive evidence that oxidative stress was present in glioma tissues in contrast with normal tissues. Our results lead to the hypothesis that a decreased capacity for oxidative stress and lower expression of genes linked to stress are associated with the evasion of immune surveillance by tumors and the advancement of cancer. *In vitro* wound healing and transwell assays confirmed that NECAP2 promotes glioma cell migration and invasion.

In addition, new evidence suggests that immunotherapy is emerging as a powerful modality for LGG patients. As a result, there is an immediate and critical need in clinical practice to uncover novel immune-related biological markers. We employed CIBERSORT and ESTIMATE techniques to examine the link between NECAP2 and cancer immune infiltration. This allowed us to thoroughly investigate the immunological significance of NECAP2 in the TCGA-LGG data. The expression level of NECAP2 correlated with the infiltration level of immune cells, including neutrophils, CD8^+^ T cells, macrophages, CD4^+^ T cells, and B cells. Therefore, NECAP2 strongly correlates with the overall immune infiltration level of glioma and can independently serve as a prognostic biological marker for glioma patients. However, it is noteworthy that the high expression of NECAP2 seems to be inversely correlated with the infiltration of Treg cells, which are widely considered to be the main immunosuppressive cells and closely associated with tumor development, contradicting the promotion of tumor development by NECAP2. Therefore, we make a reasonable speculation that a large number of infiltrating macrophages may be predominantly of the inflammatory type (M2), and NECAP2 may exert local immunosuppressive effects mainly through this pathway. Our findings suggest that NECAP2-related differences in immune infiltration may provide a convenient method for assessing the prognosis of LGG patients, further suggesting that NECAP2 may have an integral function in TME.

Our study has several limitations. First, our research relies primarily on bioinformatics techniques, and most of the data were extracted from openly available sources. Even though these findings were confirmed using cellular assays, we require clinical studies with larger sample sizes to determine our findings' reliability. Second, we conducted an in-depth investigation of the immunological function of NECAP2 in LGG; however, the mechanism that links NECAP2 to immunological responses is not completely understood. In-depth experimental research focused on cell and animal investigations to investigate the mechanistic basis of NECAP2 in LGG is required. Third, we used bioinformatics to analyze the correlation of NECAP2 with multiple TIICs and preliminary qRT-PCR experiments to examine the variations of oxidative stress genes in NECAP2-overexpressing cells. These findings must be validated using several clinical samples and coculture experimental data. Taken together, our results indicate that NECAP2 might interface with components of the TME, take part in various cancer-related, oxidative stress-related, and immune-related pathways, and contribute to the advancement of LGG. The prediction of prognosis by NECAP2-related nomogram also showed a good function. Our findings could provide foundational data that can be used clinically and in medical decision-making. More follow-up research on a larger scale is required to validate these findings.

## Figures and Tables

**Table 1:** 

Gene	Forward primer sequence (5-3)	Reverse primer sequence (5-3)
HIF1AN	TTGAGAATGAGGAGCCTGTGG	TGGGTAGAGGCACTCGAACT
HIF3A	GCCCTTTCCTGTGGAGTCAT	GAGCAGTTCAGCACCTTCCA
CUL2	ACCGGAGGGTGTGTGTTGG	CCACATATCCAATGCTAGCTCTCCT
CREBBP	AAAGCCTGCCAAGCCATCCT	CCTCATCTGCTGGTGGGTTT
EP300	ACCAGGAATGACTTCTAGTTTGA	GTGAAGGCTGCACTTGTTGG
PSMC5	TGAGTGAGGGAAGCGATGG	CCCGCAATAGGCGAACTTT
GAPDH	AATGGGCAGCCGTTAGGAAA	GCCCAATACGACCAAATCAGAG

**Table 2:** GO enrichment analysis of hub coexpressed differentially genes of NECAP2.

ONTOLOGY	ID	Description	*p* value	*p*.adjust	*q* value
BP	GO:0042391	Regulation of membrane potential	2.37e-16	6.27e-13	4.99e-13
BP	GO:0060078	Regulation of postsynaptic membrane potential	3.23e-11	4.27e-08	3.40e-08
BP	GO:0046717	Acid secretion	9.18e-11	8.10e-08	6.45e-08
BP	GO:0023061	Signal release	1.17e-09	7.73e-07	6.15e-07
BP	GO:0050890	Cognition	1.53e-09	8.10e-07	6.44e-07
CC	GO:0097060	Synaptic membrane	4.55e-15	1.23e-12	8.48e-13
CC	GO:0034702	Ion channel complex	1.37e-13	1.85e-11	1.27e-11
CC	GO:1902495	Transmembrane transporter complex	5.73e-13	5.17e-11	3.56e-11
CC	GO:1990351	Transporter complex	9.17e-13	6.21e-11	4.27e-11
CC	GO:0045211	Postsynaptic membrane	4.90e-12	2.65e-10	1.82e-10
MF	GO:0022839	Ion gated channel activity	9.31e-18	3.66e-15	2.77e-15
MF	GO:0022836	Gated channel activity	1.83e-17	3.66e-15	2.77e-15
MF	GO:0005216	Ion channel activity	2.59e-16	3.45e-14	2.61e-14
MF	GO:0022838	Substrate-specific channel activity	5.37e-16	5.37e-14	4.06e-14
MF	GO:0030594	Neurotransmitter receptor activity	8.65e-16	6.92e-14	5.23e-14

**Table 3:** KEGG enrichment analysis of hub coexpressed differentially genes of NECAP2.

ONTOLOGY	ID	Description	*p*.adjust	*q* value
KEGG	hsa04080	Neuroactive ligand-receptor interaction	1.32e-12	1.15e-12
KEGG	hsa05033	Nicotine addiction	1.34e-06	1.16e-06
KEGG	hsa04024	cAMP signaling pathway	7.29e-05	6.35e-05
KEGG	hsa04020	Calcium signaling pathway	1.58e-04	1.38e-04
KEGG	hsa04742	Taste transduction	2.15e-04	1.87e-04

**Table 4:** GSEA enrichment analysis of NECAP2.

ID	ES	NES	Adj.*p*	*q*
Biocarta_classic_pathway	0.918483	2.057596	0.019672	0.014548
Biocarta_comp_pathway	0.839301	2.025765	0.019672	0.014548
Biocarta_IL2RB_pathway	0.731654	2.045882	0.019672	0.014548
Biocarta_inflam_pathway	0.785136	2.048448	0.019672	0.014548
KEGG_allograft_rejection	0.860903	2.40242	0.019672	0.014548
KEGG_antigen_processing_and_presentation	0.637569	2.047642	0.019672	0.014548
KEGG_asthma	0.867885	2.295695	0.019672	0.014548
KEGG_autoimmune_thyroid_disease	0.754604	2.248663	0.019672	0.014548
KEGG_calcium_signaling_pathway	-0.598	-2.17971	0.020119	0.014879
KEGG_cardiac_muscle_contraction	-0.65941	-2.11927	0.019672	0.014548
KEGG_complement_and_coagulation_cascades	0.704169	2.196542	0.019672	0.014548
KEGG_cytokine_cytokine_receptor_interaction	0.598916	2.193712	0.019672	0.014548
KEGG_graft_versus_host_disease	0.823343	2.302266	0.019672	0.014548
KEGG_hematopoietic_cell_lineage	0.680197	2.195022	0.019672	0.014548
KEGG_intestinal_immune_network_for_IGA_production	0.748674	2.194467	0.019672	0.014548
KEGG_leishmania_infection	0.728146	2.274602	0.019672	0.014548
KEGG_long_term_potentiation	-0.6343	-2.01238	0.019672	0.014548
KEGG_neuroactive_ligand_receptor_interaction	-0.60349	-2.31039	0.021472	0.01588
KEGG_primary_immunodeficiency	0.725532	2.024656	0.019672	0.014548
KEGG_systemic_lupus_erythematosus	0.669466	2.279539	0.019672	0.014548
KEGG_toll_like_receptor_signaling_pathway	0.611449	2.029861	0.019672	0.014548
KEGG_viral_myocarditis	0.659361	2.048713	0.019672	0.014548
PID_FRA_pathway	0.741953	2.07468	0.019672	0.014548
PID_integrin2_pathway	0.758367	2.006002	0.019672	0.014548
PID_UPA_UPAR_pathway	0.729663	2.096119	0.019672	0.014548
Reactome_acetylcholine_neurotransmitter_release_cycle	-0.90764	-2.18144	0.019672	0.014548
Reactome_activation_of_NMDA_receptors_and_postsynaptic_events	-0.64679	-2.16665	0.019693	0.014564
Reactome_amine_ligand_binding_receptors	-0.68837	-2.00739	0.019672	0.014548
Reactome_antigen_activates_B_cell_receptor_BCR_leading_to_generation_of_second_messengers	0.831697	2.703527	0.019672	0.014548
Reactome_antigen_processing_cross_presentation	0.64507	2.137091	0.019672	0.014548
Reactome_assembly_and_cell_surface_presentation_of_NMDA_receptors	-0.68543	-2.00084	0.019672	0.014548
Reactome_binding_and_uptake_of_ligands_by_scavenger_receptors	0.853394	2.818702	0.019672	0.014548
Reactome_cardiac_conduction	-0.61844	-2.19008	0.019792	0.014637
Reactome_CD22_mediated_BCR_regulation	0.897477	2.749472	0.019672	0.014548
Reactome_cell_surface_interactions_at_the_vascular_wall	0.731733	2.601214	0.019672	0.014548
Reactome_chemokine_receptors_bind_chemokines	0.689734	2.08959	0.019672	0.014548
Reactome_cholesterol_biosynthesis	-0.76454	-2.01669	0.019672	0.014548
Reactome_complement_cascade	0.832182	2.778721	0.019672	0.014548
Reactome_costimulation_by_the_CD28_family	0.668191	2.092037	0.019672	0.014548
Reactome_creation_of_C4_and_C2_activators	0.901119	2.818541	0.019672	0.014548
Reactome_DAP12_interactions	0.718391	2.048197	0.019672	0.014548
Reactome_dopamine_neurotransmitter_release_cycle	-0.8852	-2.2751	0.019672	0.014548
Reactome_FC_epsilon_receptor_FCERI_signaling	0.707712	2.504522	0.019672	0.014548
Reactome_FCERI_mediated_CA_2_mobilization	0.848382	2.757764	0.019672	0.014548
Reactome_FCERI_mediated_MAPK_activation	0.844209	2.753048	0.019672	0.014548
Reactome_FCERI_mediated_NF_KB_activation	0.785164	2.676452	0.019672	0.014548
Reactome_FCGAMMA_receptor_FCGR_dependent_phagocytosis	0.760361	2.613884	0.019672	0.014548
Reactome_FCGR_activation	0.907847	2.831879	0.019672	0.014548
Reactome_FCGR3A_mediated_IL10_synthesis	0.813986	2.685926	0.019672	0.014548
Reactome_GABA_receptor_activation	-0.65274	-2.01773	0.019672	0.014548
Reactome_GABA_synthesis_release_reuptake_and_degradation	-0.86835	-2.13064	0.019672	0.014548
Reactome_generation_of_second_messenger_molecules	0.76005	2.125284	0.019672	0.014548
Reactome_glutamate_neurotransmitter_release_cycle	-0.90264	-2.34566	0.019672	0.014548
Reactome_immunoregulatory_interactions_between_a_lymphoid_and_a_non_lymphoid_cell	0.757493	2.680692	0.019672	0.014548
Reactome_initial_triggering_of_complement	0.887588	2.835415	0.019672	0.014548
Reactome_interaction_between_L1_and_ankyrins	-0.74173	-2.02231	0.019672	0.014548
Reactome_interferon_alpha_beta_signaling	0.67639	2.115626	0.019672	0.014548
Reactome_interferon_gamma_signaling	0.727465	2.384292	0.019672	0.014548
Reactome_interferon_signaling	0.610796	2.168773	0.019672	0.014548
Reactome_interleukin_10_signaling	0.785795	2.294225	0.019672	0.014548
Reactome_interleukin_4_and_interleukin_13_signaling	0.69424	2.309719	0.019672	0.014548
Reactome_long_term_potentiation	-0.8465	-2.17564	0.019672	0.014548
Reactome_negative_regulation_of_NMDA_receptor_mediated_neuronal_transmission	-0.83375	-2.07567	0.019672	0.014548
Reactome_netrin_1_signaling	-0.69896	-2.06518	0.019672	0.014548
Reactome_neurexins_and_neuroligins	-0.81304	-2.47068	0.019672	0.014548
Reactome_neuronal_system	-0.71786	-2.86596	0.022109	0.016351
Reactome_neurotransmitter_receptors_and_postsynaptic_signal_transmission	-0.65963	-2.44147	0.020245	0.014972
Reactome_neurotransmitter_release_cycle	-0.84341	-2.51808	0.019672	0.014548
Reactome_neutrophil_degranulation	0.529021	2.039179	0.019672	0.014548
Reactome_norepinephrine_neurotransmitter_release_cycle	-0.8668	-2.10513	0.019672	0.014548
Reactome_parasite_infection	0.8163	2.736678	0.019672	0.014548
Reactome_PD_1_signaling	0.86048	2.202703	0.019672	0.014548
Reactome_phase_0_rapid_depolarisation	-0.75541	-2.2029	0.019672	0.014548
Reactome_potassium_channels	-0.7179	-2.44077	0.019693	0.014564
Reactome_protein_protein_interactions_at_synapses	-0.77769	-2.55181	0.019792	0.014637
Reactome_regulation_of_TLR_by_endogenous_ligand	0.831026	2.005794	0.019672	0.014548
Reactome_role_of_LAT2_NTAL_lab_on_calcium_mobilization	0.893071	2.793366	0.019672	0.014548
ReactomE_role_of_phospholipids_in_phagocytosis	0.849992	2.728011	0.019672	0.014548
Reactome_scavenging_of_heme_from_plasma	0.899572	2.806068	0.019672	0.014548
Reactome_serotonin_neurotransmitter_release_cycle	-0.90522	-2.19841	0.019672	0.014548
Reactome_signaling_by_interleukins	0.548188	2.104243	0.019672	0.014548
Reactome_signaling_by_the_B_cell_receptor_BCR_	0.707585	2.47247	0.019672	0.014548
Reactome_TCR_signaling	0.596009	2.00744	0.019672	0.014548
Reactome_trafficking_of_AMPA_receptors	-0.83073	-2.26497	0.019672	0.014548
Reactome_transcriptional_regulation_by_MECP2	-0.70431	-2.18101	0.019672	0.014548
Reactome_transmission_across_chemical_synapses	-0.70329	-2.69456	0.021328	0.015773
Reactome_unblocking_of_NMDA_receptors_glutamate_binding_and_activation	-0.84303	-2.09878	0.019672	0.014548
Reactome_voltage_gated_potassium_channels	-0.81941	-2.38951	0.019672	0.014548
SA_MMP_cytokine_connection	0.897826	2.043935	0.019672	0.014548
WP_allograft_rejection	0.764972	2.503439	0.019672	0.014548
WP_calcium_regulation_in_the_cardiac_cell	-0.57617	-2.06166	0.020119	0.014879
WP_cancer_immunotherapy_by_PD1_blockade	0.809919	2.044757	0.019672	0.014548
WP_complement_activation	0.827647	2.065789	0.019672	0.014548
WP_complement_and_coagulation_cascades	0.675819	2.049387	0.019672	0.014548
WP_cytokines_and_inflammatory_response	0.786638	2.013677	0.019672	0.014548
WP_disruption_of_postsynaptic_signalling_by_CNV	-0.76751	-2.11169	0.019672	0.014548
WP_ebola_virus_pathway_on_host	0.611305	2.08446	0.019672	0.014548
WP_fibrin_complement_receptor_3_signaling_pathway	0.761487	2.173688	0.019672	0.014548
WP_fragile_x_syndrome	-0.61454	-2.1416	0.019717	0.014582
WP_GABA_receptor_signaling	-0.82265	-2.24294	0.019672	0.014548
WP_human_complement_system	0.668941	2.207294	0.019672	0.014548
WP_IL1_and_megakaryocytes_in_obesity	0.80025	2.041589	0.019672	0.014548
WP_inflammatory_response_pathway	0.795599	2.13778	0.019672	0.014548
WP_interactions_between_immune_cells_and_micrornas_in_tumor_microenvironment	0.759085	2.157469	0.019672	0.014548
WP_MBDNF_and_PROBDNF_regulation_of_GABA_neurotransmission	-0.70677	-2.01946	0.019672	0.014548
WP_microglia_pathogen_phagocytosis_pathway	0.870933	2.483106	0.019672	0.014548
WP_monoamine_GPCRS	-0.77626	-2.13576	0.019672	0.014548
WP_pathogenesis_of_SARSCOV2_mediated_by_NSP9NSP10_complex	0.813717	2.018234	0.019672	0.014548
WP_phosphodiesterases_in_neuronal_function	-0.66957	-2.01416	0.019672	0.014548
WP_plateletmediated_interactions_with_vascular_and_circulating_cells	0.881548	2.060186	0.019672	0.014548
WP_regulation_of_tolllike_receptor_signaling_pathway	0.614129	2.115056	0.019672	0.014548
WP_RETT_syndrome_causing_GEnES	-0.69881	-2.05481	0.019672	0.014548
WP_splicing_factor_nova_regulated_synaptic_proteins	-0.68675	-2.00266	0.019672	0.014548
WP_synaptic_vesicle_pathway	-0.84418	-2.52036	0.019672	0.014548
WP_tolllike_receptor_signaling_pathway	0.614678	2.036027	0.019672	0.014548
WP_type_II_interferon_signaling_IFNG	0.752447	2.104023	0.019672	0.014548
WP_tyrobp_causal_network	0.867571	2.657854	0.019672	0.014548
WP_viral_acute_myocarditis	0.644616	2.0954	0.019672	0.014548
WP_vitamin_dsensitive_calcium_signaling_in_depression	-0.70476	-2.03847	0.019672	0.014548

**Table 5:** Clinical characteristics of the glioma patients.

Characteristic	Low expression of NECAP2	High expression of NECAP2	*p*
*n*	264	264	
WHO grade, *n* (%)			0.017
G2	128 (27.4%)	96 (20.6%)	
G3	111 (23.8%)	132 (28.3%)	
IDH status, *n* (%)			< 0.001
WT	25 (4.8%)	72 (13.7%)	
Mut	238 (45.3%)	190 (36.2%)	
1p/19q codeletion, *n* (%)			< 0.001
Codel	170 (32.2%)	1 (0.2%)	
Non-codel	94 (17.8%)	263 (49.8%)	
Age, median (IQR)	41 (33, 53)	40 (31, 52)	0.156

**Table 6:** Logistic analysis of the association between, NECAP2 expression and clinical characteristics.

Characteristics	Total(*N*)	Odds ratio(OR)	*p* value
WHO grade (G3 vs. G2)	467	1.586 (1.101-2.289)	0.013
1p/19q codeletion (noncodel vs. codel)	528	475.638 (104.499-8421.042)	<0.001
Primary therapy outcome (PR&CR vs. PD&SD)	458	0.885 (0.612-1.280)	0.517
Histological type (Oligoastrocytoma and Oligodendroglioma vs. astrocytoma)	528	0.156 (0.104-0.232)	<0.001
Laterality (right vs. left)	517	0.863 (0.611-1.219)	0.403
IDH status (Mut vs. WT)	525	0.277 (0.167-0.448)	<0.001
Gender (male vs. female)	528	1.338 (0.949-1.888)	0.097
Age (>40 vs. <=40)	528	0.941 (0.669-1.324)	0.728

**Table 7:** Univariate and multivariate Cox regression analyses of clinical characteristics associated with overall survival.

Characteristics	Total(*N*)	Univariate analysis		Multivariate analysis	
		HR (95% CI)	*p*	HR (95% CI)	*p*
WHO grade	466				
G2	223	Reference			
G3	243	3.059 (2.046-4.573)	**<0.001**	2.162 (1.373-3.405)	**<0.001**
1p/19q codeletion	527				
Codel	170	Reference			
Noncodel	357	2.493 (1.590-3.910)	**<0.001**	0.803 (0.385-1.674)	0.558
Primary therapy outcome	457				
PD	110	Reference			
SD	146	0.439 (0.292-0.661)	**<0.001**	0.509 (0.314-0.823)	**0.006**
PR	64	0.175 (0.076-0.402)	**<0.001**	0.196 (0.071-0.544)	**0.002**
CR	137	0.122 (0.056-0.266)	**<0.001**	0.175 (0.079-0.388)	**<0.001**
IDH status	524				
WT	97	Reference			
Mut	427	0.186 (0.130-0.265)	**<0.001**	0.362 (0.217-0.603)	**<0.001**
Gender	527				
Female	238	Reference			
Male	289	1.124 (0.800-1.580)	0.499		
Age	527				
< =40	264	Reference			
> 40	263	2.889 (2.009-4.155)	**<0.001**	2.819 (1.770-4.491)	**<0.001**
NECAP2	527	1.848 (1.362-2.508)	**<0.001**	2.394 (1.427-4.017)	**<0.001**

## Data Availability

The datasets used and/or analyzed during the current study are available from the corresponding author on reasonable request.

## Abbreviations

UCSC: University of California Santa Cruz
GDC: Genomic data commons
ATCC: American type culture collection
BLCA: Bladder urothelial carcinoma
CHOL: Cholangio carcinoma
THCA: Thyroid carcinoma
PRAD: Prostate adenocarcinoma
LUSC: Lung squamous cell carcinoma
LUAD: Lung adenocarcinoma
LIHC: Liver hepatocellular carcinoma
KIRP: Kidney renal papillary cell carcinoma
STAD: Stomach adenocarcinoma
KIRC: Kidney renal clear cell carcinoma
KICH: Kidney chromophobe
HNSC: Head and neck squamous cell carcinoma
MF: Molecular function
BP: Biological process
CC: Cellular component.
